# Is insulin resistance a predictor for complete response in breast cancer patients who underwent neoadjuvant treatment?

**DOI:** 10.1186/s12957-020-02019-y

**Published:** 2020-09-09

**Authors:** Ozkan Alan, Tugba Akin Telli, Bilge Aktas, Sinan Koca, Ilker Nihat Ökten, Rahib Hasanov, Tugba Basoglu, Rukiye Arikan, Nazim Can Demircan, Ozlem Ercelep, Serap Kaya, Mustafa Umit Ugurlu, Handan Kaya, Nalan Akgul Babacan, Faysal Dane, Perran Fulden Yumuk

**Affiliations:** 1 Division of Medical Oncology, Marmara University School of Medicine, Marmara University Pendik Education and Research Hospital, Fevzi Cakmak Mah, Muhsin Yazicioglu C, No 10, Ust Kaynarca, 34890 Istanbul, Turkey; 2 Division of Medical Oncology, Medeniyet University School of Medicine, Istanbul, Turkey; 3 Department of General Surgery, Marmara University School of Medicine, Istanbul, Turkey; 4 Department of Pathology, Marmara University School of Medicine, Istanbul, Turkey

**Keywords:** Breast cancer, Neoadjuvant treatment, Insulin resistance, HOMA-IR, Inflammation-based indices, Pathological complete response

## Abstract

**Purpose:**

Neoadjuvant chemotherapy is the standard front-line treatment modality in locally advanced breast cancer. Achieving pathological complete response (pCR) is a significant prognostic factor for prolonged disease-free and overall survival. Insulin resistance is defined as a pathological condition in which insulin effect is impaired in peripheral target tissues such as the skeletal muscle, liver, and adipose tissue. The relationship between breast cancer and insulin resistance is controversial. In this study, our aim is to evaluate the role of insulin resistance, body mass index (BMI), metabolic syndrome, and inflammation markers to predict complete response in breast cancer patients who underwent neoadjuvant treatment.

**Methods:**

Data from 55 locally advanced non-diabetic breast cancer patients, treated with neoadjuvant chemotherapy between 2015 and 2017, were retrospectively evaluated. Homeostatic model assessment, IR = insulin resistance (HOMA-IR) was calculated by using the obtained insulin and fasting blood glucose values before neoadjuvant chemotherapy (fasting insulin × fasting glucose/405). We considered a cut-off of 2.5 for insulin resistance. The systemic inflammatory index (SII), neutrophil-lymphocyte ratio (NLR), and platelet-lymphocyte ratio (PLR) were calculated.

**Results:**

Twenty-five patients had no insulin resistance. The most common pathologic subtype (56%) was hormone receptor (HR) positive and human epidermal growth factor receptor-2 (Her-2)-negative invasive ductal carcinoma. Sixteen (29%) patients had a pathological complete response (pCR). We found that the probability of pCR in patients with insulin resistance was 4.7 times lower than that in patients without insulin resistance [OR: 4.7 (95%CI 1.7–17.2), *p* = 0.01].

**Conclusion:**

Our results revealed that insulin resistance may have a negative effect on pathological complete response (pCR) following neoadjuvant therapy particularly with hormone-positive and Her-2-negative cases of non-diabetic breast cancer.

## Background

Worldwide, breast cancer is the most commonly diagnosed cancer and is the second most common cause of cancer-related death in women [1]. Current-tailored treatment of breast cancer is discussed in multidisciplinary teams which involved surgical oncology, radiation oncology, and medical oncology for each patient, and this approach was reported to increase breast cancer survival rates [2]. Nowadays, neoadjuvant chemotherapy (NAC) has become a standard treatment approach in breast cancer patients with locally advanced disease [3]. Neoadjuvant therapy has some advantages such as downstaging the tumor, giving a chance to convert inoperable tumors to operable tumors or to undergo breast-conserving surgery instead of mastectomy, and evaluate tumors’ biological behavior. Pathologic complete response (pCR) after NAC is a significant surrogate prognostic marker for both disease-free and overall survival in breast cancer [4, 5]. Expected pCR with NAC is approximately 30% in HER2-positive and 30 to 50% in triple-negative histology, whilst it is less than 10% in hormone-positive and HER2-negative breast cancer patients [6–8].

Insulin is an anabolic hormone that regulates blood glucose homeostasis. It is produced by beta-cells of the islets of Langerhans in the pancreas. Insulin resistance is a pathological condition characterized by disruption of insulin effects in peripheral tissues including the skeletal muscle, liver, and adipose tissue [9]. Insulin resistance is a typical characteristic of most patients with type 2 diabetes mellitus. Also, it is closely linked to obesity and metabolic syndrome. Obesity and its induced inflammation have a major role in the pathogenesis of insulin resistance. It is stated in recent studies that there is an association between decreased insulin sensitivity and chronic inflammation in people with obesity and insulin resistance. Increased levels of proinflammatory cytokines such as tumor necrosis factor-α, interleukin-6, and interleukin-8 were documented in people with diabetes or insulin resistance [10–12]. Also, several meta-analysis and studies have reported that insulin resistance may be related to increased breast cancer risk and worse prognosis. In an epidemiologic study done on breast cancer survivors by Godwin et al., an association between high-fasting insulin levels and two-fold breast cancer recurrence and three-fold cancer-related death risk increase was shown [13–17]. However, pathophysiological mechanisms underlying the relationship between insulin resistance and cancer development, and prognosis are not fully understood. In vitro and animal studies, suggested elevated levels of insulin-like growth factor-1 (IGF-1) induced by hyperinsulinemia to be the main factor in cancer development and progression [18–20].

To the best of our knowledge, there is no evidence of an association between insulin resistance and pCR in locally advanced breast cancer patients who underwent NAC in the medical literature. Based on this hypothesis, we aim to evaluate the role of insulin resistance to predict pCR in non-diabetic breast cancer patients who underwent NAC.

## Patients and methods

### Patients

We designed a prospective study that evaluated the significance of immune-profiling in locally advanced and metastatic breast cancer patients in 2015. One hundred-twelve patients were included. This study is ongoing. We retrospectively performed a subgroup analysis in this study cohort. The data from a total of 55 locally advanced breast cancer patients treated with NAC between 2015 and 2017 in Marmara University Medical Oncology Outpatient Clinic were evaluated. Inclusion criteria were histologically/cytologically diagnosis of breast cancer, having locally advanced disease, treated with NAC, and having complete medical records. Since type 2 diabetes mellitus could be a confounding factor that can affect the results of pCR, patients with the diagnosis of diabetes mellitus were excluded from the current study. All patients were clinically staged by contrast-enhanced breast MRI and PET/CT before neoadjuvant treatment. All patients followed-up in our outpatient clinic until November 2019, and this was the time frame for data cutoff for survival analysis.

### Blood analysis

All routine peripheral venous blood samples were obtained from patients on an empty stomach early in the morning a day before NAC. Data of insulin level, fasting glucose level, lipid profile, number of white blood cells (WBC), sedimentation rate, and C-reactive protein (CRP) level were recorded from the medical database. The systemic inflammatory index (SII), neutrophil-lymphocyte ratio (NLR), and platelet-lymphocyte ratio (PLR), which are indicators of inflammation, were calculated as follows: SII = (neutrophile counts × platelet counts)/lymphocyte counts, NLR = neutrophile count/lymphocyte count, and PLR = platelet count/lymphocyte count.

### Homeostatic model assessment, IR = insulin resistance (HOMA-IR)

HOMA-IR was calculated as follows: (fasting insulin level × fasting blood glucose level)/405. We accepted the cutoff value of HOMA-IR as > 2.5 to define insulin resistance [21]. The study cohort was categorized as patients with and without insulin resistance.

### Metabolic syndrome

The National Cholesterol Education Program (NCEP) Adult Treatment Panel III (ATP III) criteria have been used for the diagnosis of metabolic syndrome [22].

### Neoadjuvant chemotherapy regimens

NAC was performed according to the following protocols, regardless of tumor hormonal status:

A. Her-2-positive patients: AC regimen (Doxorubicin 60 mg/m^2^ plus cyclophosphamide 600 mg/m^2^, on day 1, and repeated in every 21 days) for 4 cycles; followed by weekly paclitaxel dose of 80 mg/m^2^ for 12 weeks and trastuzumab 8 mg/kg loading, and 6 mg/kg maintenance dose, repeated in every 3 weeks. Also, trastuzumab was continued for a total of 1 year after breast surgery. Pertuzumab was not used in the neoadjuvant chemotherapy regimen because it is not approved and reimbursed at that time in Turkey.
B. Her-2-negative patients: Same dose and schedule of AC regimen followed by weekly paclitaxel for a total of 12 weeks.

All patients completed the full course of chemotherapy regimens. All patients with estrogen and/or progesterone (HR)-positive patients received appropriate anti-hormonal treatments (tamoxifen for pre-menopausal and aromatase inhibitors for post-menopausal patients).

### Pathologic evaluation

All patients in the study cohort were diagnosed with invasive breast cancer by tru-cut biopsy. Immunohistochemical (IHC) staining was used to determine estrogen receptor (ER), progesterone receptor (PR), and Ki67. Detection of strong positive (+3) membranous staining for human epidermal growth factor receptor 2 (HER-2) by IHC was accepted as HER-2-positive. Fluorescence in situ hybridization analysis (FISH) was performed in samples that had moderate (2+) membranous staining in IHC, and those which showed amplification by FISH were considered as HER-2-positive. Ki67 ≥ 15% was considered as having a high proliferative index. All patients were operated within an average of 4–10 weeks after NAC by expert breast surgeons. Pathologic complete response (pCR) was defined as no tumor tissue or isolated tumor cells detectable in pathology specimen (both in breast and lymph nodes) after surgery [23–25].

### Statistical analysis

Stratification was performed according to the status of insulin resistance (present or absent). Comparison of categorical variables was carried out using the Pearson *χ*^2^ test. Univariate and multivariate logistic regression models were conducted to assess factors to pCR. Disease-free survival time (DFS) was defined as the time from breast surgery (the first date of no disease) until radiological progression, death, or last visit date. Overall survival time (OS) was defined as the time from the start of systemic treatment until death with any reason or last visit. Survival analysis was done with the Kaplan-Meier method. Odds ratio (OR) with 95% confidence intervals (CI) were also calculated. All *p* values were 2-sided in the tests, and *p* values of < 0.05 were considered statistically significant. SPSS 22 program was used for statistical analysis.

## Results

A total of 55 female patients were included in the study. The median age was 48.5 years (min 27-max 80) and 52% of the patients had clinical stage III disease. Thirty (54.5%) patients had insulin resistance. Patients with insulin resistance had a higher body mass index (BMI), triglyceride, and fasting glucose level compared to patients without insulin resistance. Median BMI was 33.25 (range 18.9–43.4) in patients with an insulin resistance group, and 27.01 (range 21–35.2) in the group without insulin resistance (*p* = 0.01). The median triglyceride and fasting glucose levels were 121 mg/dl (range 58–467) and 103 mg/dl (range 82–125) in patients with an insulin resistance group, and 89 mg/dl (range 56–164) and 91 mg/dl (range 72–115) (*p* = 0.007, 0.003, respectively). Seventeen (57%) patients with insulin resistance also had metabolic syndrome. There was a statistically significant difference between the two groups (*p* = 0.006). The most common pathologic subtype was invasive ductal carcinoma, and 56% of all patients had HR-positive—HER-2-negative disease. There was no difference in terms of pathologic subtypes and receptor status between the two groups. Patients having high Ki-67 values were statistically higher in the cohort without insulin resistance (*p* = 0.01). Baseline demographic and clinicopathologic findings were outlined in Table 1.

**Table 1:** Baseline demographic and clinicopathologic findings

		All patients (***n*** = 55)	Insulin resistance		*p*
			Yes (***n*** = 30)	No (***n*** = 25)	
**Age (median) (min-max)**		48.5 (27–80)	52 (29–80)	45 (27–66)	0.06
**BMI (median) (min-max)**		29.6 (18.9–43.4)	33.25 (18.92–43.4)	27.01 (21–35.2)	0.01
**Waist circumference (cm)**		100 (56–116)	100 (56–116)	89 (56–106)	0.7
**Hip circumference (cm)**		108 (58–134)	110 (58–134)	106 (95–120)	0.5
**Fasting glucose level (mg/dl)**		99 (72–125)	103 (82–125)	91 (72–115)	0.003
**HDL (mg/dl)**		57 (24–96)	55 (24–83)	59 (40–96)	0.07
**LDL (mg/dl)**		124 (51–219)	120 (51–198)	139 (61–219)	0.6
**Total cholesterol (mg/dl)**		215 (117–314)	205 (117–298)	218 (127–314)	0.5
**Trigliseride (mg/dl)**		98 (56–467)	121 (58–467)	89 (56–164)	0.007
**Metabolic syndrome**	Yes	22 (40%)	17 (57%)	5 (20%)	0.006
	No	33 (60%)	13 (43%)	20 (80%)	
**Menopause status (*n*) (%)**	Pre-menopause	32 (58%)	16 (53%)	16 (69%)	0.4
	Post-menopause	23 (42%)	14 (47%)	9 (31%)	
**Clinical stage**	2A	9 (16%)	7 (23%)	2 (8%)	0.2
	2B	18 (32%)	10 (33%)	8 (32%)	
	3A	20 (36%)	8 (27%)	12 (48%)	
	3B	7 (14%)	5 (17%)	2 (8%)	
	3C	1 (2%)	0 (0%)	1 (4%)	
**Pathologic subtype**	Invazive ductal	44 (80%)	26 (87%)	18 (72%)	0.8
	Invazive lobular	5 (9%)	1 (3%)	4 (16%)	
	Others	6 (11%)	3 (10%)	3 (12%)	
**Receptor status**	HR+, HER-2−	31 (56%)	17 (56%)	14 (56%)	0.7
	HR−, HER-2+	7 (13%)	5 (16%)	2 (8%)	
	HR+, HER-2+	9 (16%)	4 (14%)	5 (20%)	
	HR−, HER-2−	8 (15%)	4 (14%)	4 (16%)	
**Hormone receptor status**	ER + PR+	31 (56%)	18 (60%)	13 (52%)	0.9
	ER + PR−	9 (16%)	3 (10%)	6 (24%)	
	ER − PR+	0 (0%)	0 (0%)	0 (0%)	
**Ki 67 index**	≤ 15	13 (24%)	11 (37%)	2 (8%)	0.01
	> 15	42 (76%)	19 (63%)	23 (92%)	
**Pathological response**	CR	16 (29%)	5 (16%)	11 (44%)	0.02
	Non-CR	39 (71%)	25 (84%)	14 (56%)	

*HDL* high-density lipoprotein, *LDL* low-density lipoprotein, *HR* hormone receptor, *Her-2* human epidermal growth factor receptor 2, *ER* estrogen receptor, *PR* progesterone receptor

Inflammatory markers such as WBC, neutrophil count, and CRP were statistically significantly higher in patients with insulin resistance (*p* = 0.004, 0.005, and 0.01, respectively). Median NLR, PLR, and SII were calculated as 3.3, 225.3, and 963 in the whole cohort. Median NLR and SII were 3.6 and 999 in patients with insulin resistance. Patients with insulin resistance had higher SII and NLR compared to patients without insulin resistance (*p* = 0.01, 0.02, respectively). But median PLR was 230.8 in patients with insulin resistance, and there was no statistically significant difference between the two groups. Results of inflammatory markers according to insulin resistance status were shown in Table 2.

**Table 2:** Results of inflammatory markers according to insulin resistance status

	All patients (***N*** = 55)	Insulin resistance		
		Yes (***n*** = 30)	No (***n*** = 25)	*p*
**White blood cell (ц/L) (min-max)**	6700 (3100–11100)	7550 (4100–11100)	6100 (3100–8900)	0.004
**Neutrophile (ц/L) (min-max)**	4330 (1581–8991)	4805 (1827–8991)	3538 (1581–6597)	0.005
**Lymphoctye (ц/L) (min-max)**	1155 (585–2115)	1188 (640–1905)	1103 (585–2115)	0.4
**Platelet (ц/L) (min-max)**	282000 (172000–418000)	28200 (172000–41800)	262000 (174000–361000)	0.1
**Neutrophil-lymphoctye ratio (NLR) (median) (min-max)**	3.3 (min 1.9–max 7.6)	3.6 (min 2.2–7.6)	3.03 (min 1.9–max 5.2)	0.02
**Platelet-lymphoctye ratio (PLR) (median) (min-max)**	225.3 (min 101.6–max 418.8)	230.8 (min 126.8–max 390.2)	223.1 (min101.6–max 418.8)	0.8
**Systemic inflammation index (SII) (median) (min-max)**	963 (min 440–max 2458)	999 (min 511–max 2458)	884 (min 440–max 1438)	0.01
**CRP (mg/L) (median) (min-max)**	3.4 (1.59–43)	4.3 (2–43)	3.3 (1.5–12.5)	0.01

After NAC, 16 (29%) patients had pCR. Five of these patients had insulin resistance. The pCR rates according to the pathological subtypes were outlined in Table 3. The pCR rate was statistically significantly lower in patients with insulin resistance than those without insulin resistance (16% vs 44%, *p* = 0.02). The effects of BMI, presence of metabolic syndrome, inflammatory markers, pathological receptor subtype (HER-2, ER, and PR status), KI67 index, and insulin resistance on pCR have been assessed by the logistic regression. Insulin resistance and PR status were detected to be significantly associated with pCR. We found that the probability of pCR in patients with insulin resistance was 4.7 times lower than that in patients without insulin resistance [OR: 4.7, 95%CI 1.7–17.2, *p* = 0.01]. Also, the probability of pCR in patients with PR-negative tumors was determined as 3.6 times higher than that in patients with PR-positive [OR: 3.6, 95%CI 1.0–13.0, *p* = 0.04]. The pCR was obtained in 5 (55%) of 9 PR-negative patients. However, only 5 (16%) of 31 PR-positive patients had a pCR (*p* = 0.01). The logistic-regression model of factors affecting pCR was given in Table 4.

**Table 3:** The pCR rates according to the pathological subtypes

Pathologic subtype	Pathological response	
	CR (***n*** = 16)	Non-CR (***n*** = 39)
**HR+, HER-2− (*n*** **= 31)**	8 (25%)	23 (75%)
**HR+, HER-2+ (*n*** **= 9)**	2 (22%)	7 (78%)
**HR−, HER-2 + (*n*** **= 7)**	3 (42%)	4 (58%)
**HR−, HER-2− (*n*** **= 8)**	3 (38%)	5 (62%)

*HR* hormon receptor, *Her-2* human epidermal growth factor receptor 2

**Table 4:** The logistic-regression model of factors affecting pathological complete response

	***n***	Pathologic response		Univariate analysis				Multivariate analysis			
		CR ***n*** = 16 (%)	Non-CR ***n*** = 39 (%)	OR	95% CI		***p***	OR	95 % CI		***p***
					Lower	Upper			Lower	Upper	
**Body mass index (BMI)**	≤ 29.6 (*n* = 31)	9 (29)	22 (71)	1.1	0.3	3.6	0.8				
	> 29 (*n* = 24)	7 (29)	17 (71)								
**Metabolic syndrome**	Yes (*n* = 22)	5 (23)	17 (77)	1.9	0.5	6.6	0.2				
	No (*n* = 33)	11 (33)	22 (63)								
**Insulin resistance**	No (*n* = 25)	11 (44)	14 (56)	4.6	1.3	15.9	0.02	4.7	1.7	17.2	0.01
	Yes (*n* = 30)	5 (16)	25 (84)								
**NLR**	≤ 3.3 (*n* = 26)	9 (35)	17 (65)	1.9	0.6	6.2	0.8				
	> 3.3 (*n* = 29)	7 (24)	22 (76)								
**SII**	≤ 963 (*n* = 28)	10 (36)	18 (74)	2.2	0.6	7.1	0,1				
	> 963 (*n* = 27)	6 (22)	21 (78)								
**PLR**	≤ 225.3 (*n* = 27)	11 (40)	16 (60)	2.5	0.7	8.2	0.06				
	> 225.3 (*n* = 28)	5 (18)	23 (82)								
**CRP**	< 5 (mg/L) (*n* = 38)	10 (26)	28 (74)	1.3	0.3	4.5	0.6				
	≥ 5 (mg/L) (*n* = 17)	6 (35)	11 (65)								
**Ki 67 index**	≤ 15 (*n* = 13)	2 (18)	11 (82)	0.3	0.6	1.67	0.1				
	> 15 (*n* = 42)	14 (33)	28 (67)								
**Her-2 status**	Positive (*n* = 16)	7 (44)	9 (56)	0.5	0.1	1.97	0.3				
	Negative (*n* = 39)	9 (23)	30 (77)								
**Estrogen receptor status**	Positive (*n* = 40)	10 (25)	30 (75)	1.7	0.5	6.1	0.3				
	Negative (*n* = 15)	6 (40)	9 (60)								
**Progesterone receptor status**	Positive (*n* = 31)	5 (16)	26 (84)	3.5	1.0	11.6	0.01	3.6	1.0	13.0	0.04
	Negative (*n* = 9)	5 (55)	4 (45)								

The median follow-up period was 41 months (min 15–max 49). During the follow-up period, the disease recurred in seven patients, and four patients died. Median disease-free and overall survival could not be calculated by the Kaplan-Meier method because of the small number of recurrences and deaths during the short follow-up period.

## Discussion

In our study, we found that the probability of having a pathological complete response (pCR) after NAC in patients with insulin resistance was lower compared to those without insulin resistance. We detected that systemic inflammation markers were also elevated in patients with insulin resistance, but these markers were not associated with pCR. To our knowledge, this is the first study to show the relationship between insulin resistance and pCR in locally advanced breast cancer patients treated with NAC. Our findings suggested that the presence of insulin resistance could be a candidate biomarker for the pCR prediction in breast cancer patients who underwent neoadjuvant treatment.

Today, there is growing evidence showing the relationship between insulin resistance and cancer development. Some epidemiologic studies indicated that fasting insulin levels might represent a risk factor for some cancer types such as colorectal, endometrium, and breast cancer [26–28]. Insulin can bind to both insulin receptor (IR) and IGF-1 receptor (IGF-1R), and those are both tyrosine kinase transmembrane receptors. Hyperinsulinemia induces the production of insulin-like growth factor-1 (IGF-1) by the liver. IGF-1 signal pathway is contributed to cell proliferation and growth. As a result of the interaction of IGF-1 and IGF-1R, tyrosine kinase receptor activates, which evokes to induce the MAPK (mitogen-activated protein kinase) and PI3K (the phosphatidylinositol 3-kinase) pathways. While the MAPK pathway is mostly related to cell growth and proliferation, the PI3K pathway is mostly associated with metabolic and anti-apoptotic effects [29, 30]. The relationship between the IGF-1 pathway and tumor development had been shown in various cancer types such as lung, prostate, breast, gynecological, and gastrointestinal cancers [31]. Additionally, Denduluri et al. have shown that increased IGF-1 pathway activation may have promoted cancer progression and contribute to the development of resistance to treatment [32]. Ireland L.et al. have reported that 75% of breast cancer patients show insulin/IGF-1 pathway activation, and this was correlated with increased macrophage infiltration and advanced stage [33]. Also, this has been associated with an increasing mortality rate in breast cancer patients [34, 35].

According to current guidelines, NAC is the standard approach in locally advanced breast cancer patients. We know that pCR is a surrogate marker for survival in these patients’ population. The smaller tumor size, higher histological grade, Her-2 positivity, and hormonal receptor status have been determined predictive biomarkers to identify pCR in literature [36]. There is limited data on the effect of insulin resistance on the pCR in breast cancer patients receiving neoadjuvant therapy. Jiralerspong et al. have reported that a significantly higher pCR rate in diabetic breast cancer patients treated with metformin (24% versus 8%, *p* = 0.02). Additionally, they have suggested that using metformin was an independent predictive biomarker of pCR during neoadjuvant treatment (Odds ratio: 2.95, 95%CI 1.07–8.17, *p* = 0.04) [37]. Dowling et al. have explained that the mechanism of action of metformin on the pCR during the neoadjuvant treatment by two hypotheses. While it has a direct effect with mTOR pathway inhibition, it reduces circulating insulin levels and indirectly causes inhibition of the insulin signal activation pathway [38]. Bhargava et al. have reported that high IGF-1 receptor expression was related to lower pCR rate in only ER-receptor-positive breast cancer patients [39]. Similarly, another study has been shown that low IGF-1 receptor expression was related to a higher pCR rate in Her-2-positive breast cancer (OR: 3.93, 95%CI 1.13–13.63, *p* = 0.031). However, no relationship has been found between IGF-1 level and survival [40]. The current study revealed that insulin resistance was associated with a low pCR rate, similar to literature. To note, we could not demonstrate metabolic syndrome and body mass index as a predictive factor for pCR. Similar results have been indicated by Tong et al., who concluded that metabolic syndrome and body mass index were not a predictive biomarker for pCR in Her-2-positive breast cancer [40]. Furthermore, Goodwin et al. have reported that fasting insulin was associated with distant recurrence and death in early-stage breast cancer patients [17]. Likewise, Ferroni et al. have suggested that pretreatment insulin levels may have been a biomarker of poor prognosis in non-diabetic breast cancer patients [41].

The relationship between systemic inflammation and obesity, type-2 diabetes mellitus, or insulin resistance is well known in the current literature [42]. It has been reported that inflammatory markers such as CRP, fibrinogen, and IL-6 increase in insulin resistance [43, 44]. Karakaya et al. showed that the N/L ratio, which is a systemic inflammation marker, increased in insulin resistance. Also, they found that positive correlations between insulin levels and the NL ratio, WBC counts, and neutrophil counts [45]. In another study, WBC subtypes and N/L ratio were reported to be independently associated with insulin resistance [46]. Similar to literature, in our study, we found that some inflammatory markers, such as WBC, neutrophil, CRP, NL ratio, and SII, were increased in breast cancer patients with insulin resistance compared to those without insulin resistance. The NL ratio has been accepted as a predictive biomarker for prognosis in several cancers. In a meta-analysis, a high NL ratio was shown to be statistically significantly associated with poor response to NAC in breast cancer patients [47]. Rivas and colleagues have shown the NL ratio might be a predictive biomarker for tumor response in luminal B breast cancer patients receiving neoadjuvant therapy [48]. Conversely, we did not find a statistically significant association between NLR, SII, and PLR and tumor response to neoadjuvant therapy. Also, similar to our results, Suppan and colleagues could not demonstrate the predictive or prognostic value of the NL ratio in breast cancer patients with neoadjuvant treatment [49].

Our study has some limitations. Firstly, the number of patients in the study cohort was relatively small, and this situation might have caused bias. Besides, our 41 months of median follow-up period was relatively short, and therefore, median survival could not be reached yet. Unlike other studies, we included consecutive patients with insulin levels already available in their data, and we concluded that patient selection may have affected our results. Thirdly, we checked metabolic parameters only once before starting NAC. Also, serial measurements of metabolic and inflammatory parameters during follow-up could be important to elucidate the relationship between the changes of these parameters and pCR. Lastly, patients without insulin resistance had a higher Ki 67 index, and this may be a confounding factor that affects our results.

## Conclusion

Our results revealed that insulin resistance may have a negative effect on pathological complete response (pCR) following neoadjuvant therapy particularly with hormone-positive and Her-2 negative cases of non-diabetic breast cancer. We did not find inflammation markers and presence of metabolic syndrome as an independent predictive factor for pCR. Our sample size is limited, and more comprehensive, better designed, and prospective studies are needed to verify our results.

## Data Availability

All the data supporting our findings is contained within the manuscript.
